# Individualized faecal immunochemical test cut-off based on age and sex in colorectal cancer screening

**DOI:** 10.1016/j.pmedr.2021.101447

**Published:** 2021-06-09

**Authors:** Tim L. Kortlever, Manon van der Vlugt, Evelien Dekker, Patrick M.M. Bossuyt

**Affiliations:** a Department of Gastroenterology and Hepatology, Amsterdam University Medical Centers, Amsterdam, The Netherlands; b Department of Clinical Epidemiology, Biostatistics, and Bioinformatics, Amsterdam University Medical Centers, Amsterdam, The Netherlands

**Keywords:** Colorectal cancer, Screening, Faecal immunochemical test, Risk stratification

## Abstract

The risk of having colorectal cancer (CRC) or its precursors vary with age and sex. Yet, most CRC screening programs using the quantitative faecal immunochemical test (FIT) use a uniform FIT cut-off. We aimed to calculate individualized FIT cut-offs based on age and sex. Data from a study of 1,112 asymptomatic average-risk screening participants undergoing colonoscopy without preselection were used to build a logistic regression model to calculate the risk of having advanced neoplasia (AN) at colonoscopy using age, sex, and FIT concentration as variables. We calculated age- and sex-adjusted FIT cut-off concentrations based on a uniform risk threshold. In a total of 101 of the 1,112 participants AN was detected at colonoscopy. We selected a risk threshold that would produce a specificity of 96.9% in the study group, matching the specificity of FIT at a cut-off of 20 µg Hb/g faeces. At this threshold, age- and sex-adjusted FIT cut-off concentrations ranged from 36.9 µg Hb/g faeces for 50-year-old women to 9.5 µg Hb/g faeces for 75-year old men. At this level of specificity, the risk-based model reached a sensitivity for AN of 28.7% (95%CI: 20.8 to 38.2) versus 27.7% (95%CI: 19.9 to 37.1) for FIT only. Using a risk threshold instead of a uniform FIT-based threshold for inviting screening participants to follow-up colonoscopy ensures that everyone has a comparable risk of AN prior to colonoscopy and may improve the detection of advanced neoplasia, although the absolute magnitude of the increase is likely to be limited.

## Highlights

• We examined individualized Faecal Immunochemical Test (FIT) cut-offs for the detection of advanced neoplasia.
• Individual FIT cut-offs were calculated by age and sex for an overall specificity of 96.9%
• Individual FIT cut-offs at this level of specificity may vary four-fold between screening participants.
• Risk-based thresholds may improve personalization of CRC screening.

## 1 Introduction

Many screening programs worldwide use the faecal immunochemical test (FIT) to screen for colorectal cancer (CRC) (Schreuders et al., 2015). Its benefits include the ease of use, the possibility to be send by mail, and the automated and quantitative result. Participation rates in FIT-based screening programs are higher than those in colonoscopy-based programs (Quintero et al., 2012, Chen et al., 2020).

FIT-based screening also has limitations. In a screening setting where the FIT cut-off concentration is set to achieve a high specificity, the sensitivity to detect advanced adenomas (AA) is relatively low (Gies et al., 2018). In addition, even though the risk of advanced neoplasia (AN) varies between age groups and between men and women (Auge et al., 2014), FIT is commonly used at a uniform cut-off concentration. This means that two individuals of different age and/or sex, but similar FIT concentrations, have a different a priori risk of having AN at colonoscopy. For example, in a group of individuals with the same FIT concentration, the proportion of negative colonoscopies in 50-year-old women will be higher than that in 70-year-old men. In addition, older screenees who have a FIT result just below the positivity threshold will not be invited to colonoscopy, despite their absolute risk of AN being higher than that of younger screenees with a FIT result just above the positivity threshold, who are referred for colonoscopy.

There is ample data available showing the risk of CRC or advanced neoplasia (AN) increases with FIT concentration, with age, and that this risk is different for males and females (Auge et al., 2014, White et al., 2018, McDonald et al., 2011, Kolligs et al., 2011, Ferlitsch et al., 2011). A multivariable risk model might use this extra information to calculate individual FIT cut-off concentrations based on a uniform risk threshold. This ensures that everyone who is tested positive by the model has a comparable risk of AN prior to colonoscopy. Such a risk-based approach may also improve the yield of AN in screening.

To date, several risk models have been developed, all aiming to improve detection of AN in screening. One study from our group showed that a model based on the FIT result, age, sex, smoking status, calcium intake, and family history of CRC significantly improved discrimination compared to FIT alone (Stegeman et al., 2014). The downside of this model is that it requires a questionnaire to collect data, which may affect participation. Other models use an additional biomarker, which may substantially increase screening costs. In contrast, data on age and sex are generally readily available in screening programs and their use may improve screening without increasing costs or the burden on participants. To our knowledge, a simple risk model using only FIT concentration, age and sex in an average-risk population has not been reported yet.

We developed a multivariable risk model that calculates the risk of detecting AN at colonoscopy based on FIT concentration, age, and sex. With this model, we calculated age- and sex-based FIT cut-off concentrations that would yield a uniform AN risk at colonoscopy and, for the screening population, a specificity that matches that of a uniform FIT cut-off concentration of 20 µg Hb/g faeces. We also evaluated the performance of this model in detecting AN at colonoscopy, compared to FIT screening with a uniform cut-off concentration.

## 2 Methods

### 2.1 Study population

Data for this study were collected in the Colonoscopy or Colonography for Screening (COCOS) study, a population-based multicentre randomized trial described in detail elsewhere (de Wijkerslooth et al., 2010). In short, in the colonoscopy arm of the trial participants aged 50 to 75 with no history of CRC screening were invited to undergo primary colonoscopy screening and complete a FIT. Individuals with a history of inflammatory bowel disease, colorectal cancer, or colonic adenomas were excluded. All participants provided written informed consent.

### 2.2 FIT, colonoscopy, and histology

Consenting participants performed a one sample FIT (OC-Sensor, Eiken Chemical, Tokyo, Japan) 48 h before colonoscopy and its preparations. Colonoscopies were performed according to the standard quality aspects of the American Society for Gastrointestinal Endoscopy (Nagengast and Kaandorp, 2001). A low fibre diet and 2 L of hypertonic polyethylene glycol was prescribed to all participants to prepare for colonoscopy. Collected lesions were evaluated by gastrointestinal pathologists, using the Vienna criteria (Schlemper et al., 2000). AN was defined as CRC and/or an adenoma of 10 mm or larger, villous histopathology of 25% or more, or high-grade dysplasia.

### 2.3 Model development

We built a logistic regression model to calculate the risk of detecting AN at colonoscopy, using age, sex, and FIT concentration as predictors. To improve the fit of the model, the square root of the FIT concentration was added to the linear predictor for FIT. Penalized Maximum Likelihood Estimation was used to shrink the coefficients. After calculating the risk of AN with this model for all participants, we then selected a uniform risk threshold that would have the same specificity as a uniform FIT cut-off concentration of 20 µg Hb/g faeces in this study group. We chose this cut-off concentration, because it is commonly used in different screening programs around the world.

### 2.4 Statistical analysis

The generalized likelihood ratio test statistic was used to compare goodness-of-fit for a model with FIT only and the model with the other predictors. Model performance in terms of discrimination for the detection of AN was assessed using the c-statistic (equivalent to the area under the receiver operator characteristic curve, AUC). Differences in AUC were tested with the DeLong test statistic. We evaluated the clinical sensitivity of the model at different levels of specificity and cut-off concentrations, comparing it to FIT only. P-values below 0.05 were considered to indicate statistically significant differences. All analyses were performed in RStudio (version 1.2.1335) using the packages *rms*, *cutpointr*, *bootLR*, *ROCR, and ggplot2* (R Core Team, 2019, Thiele, 2020, Marill, 2017, Sing et al., 2005, Wickham, 2016, Harrell, 2019).

## 3 Results

### 3.1 Baseline characteristics

In the COCOS study, 6600 persons were invited for primary colonoscopy screening. A total of 1426 participants (22%) underwent a colonoscopy. In this group, 1112 participants also completed the FIT (80%). Their mean age was 60.6 years (SD 6.2 years); 569 (51.2%) were male. Overall, 58 (5.2%) participants had a FIT concentration equal or higher than 20 µg Hb per gram faeces.

In 101 of the 1,112 participants (9.1%) AN were detected at colonoscopy; CRC was detected in 7 participants and AA in 94. Participants with AN were significantly older (95% CI 0.7–3.2, *p* = 0.002) than those without AN. AN were detected in 56 out of 569 males (9.8%) versus 45 out of 543 females (8.3%; *p* = 0.42). Of the 101 participants with AN, 59 (58.4%) had a FIT concentration < 5 µg Hb/g faeces.

### 3.2 Individualized FIT cut-offs

In this study group, a FIT cut-off concentration of 20 µg Hb/g faeces would yield a specificity of 97.0% and a sensitivity of 27.7% in detecting AN. We calculated the risk of detecting AN at colonoscopy in all participants, based on their age, sex, and FIT concentration. We then selected the risk-based threshold that would have a specificity as close as possible to 97.0%. This turned out to be a risk of 0.2524 of detecting AN at colonoscopy, which had a specificity of 96.9%.

**Fig. 1:**
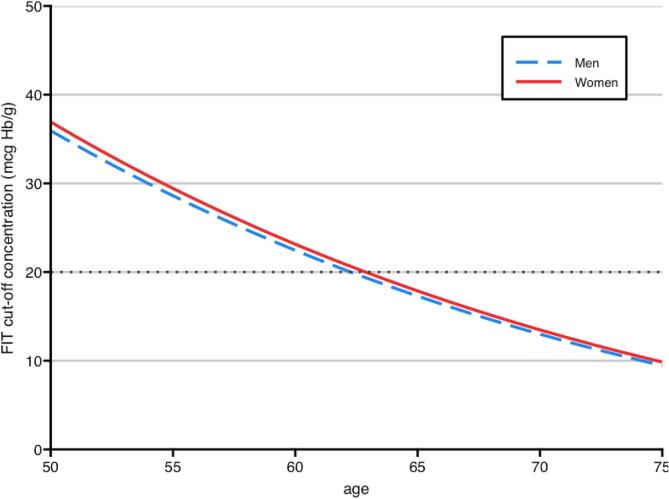
Individualized FIT cut-off concentrations at a model specificity of 96.9% (equivalent to the specificity of FIT at 20 µg Hb/g faeces).

Since this risk is calculated based on age, sex, and FIT concentration, we could then identify the FIT cut-off concentrations for each combination of age and sex corresponding to the selected risk-based threshold (Fig. 1, and Supplementary Table 1). Relying on a uniform risk threshold for screening would mean, for example, that a woman of 50 years old needs a FIT result of 36.9 µg Hb/g to achieve the same risk of having AN at colonoscopy as a 75-year-old man with a FIT result of 9.5 µg Hb/g: an almost four times higher concentration. We repeated the analysis for risk thresholds with a specificity equivalent to uniform FIT cut-off concentrations of 10, 15, and 50 µg Hb/g faeces (Supplementary Figs. 1-4 and Supplementary Table 1)

### 3.3 Model performance

**Fig. 2:**
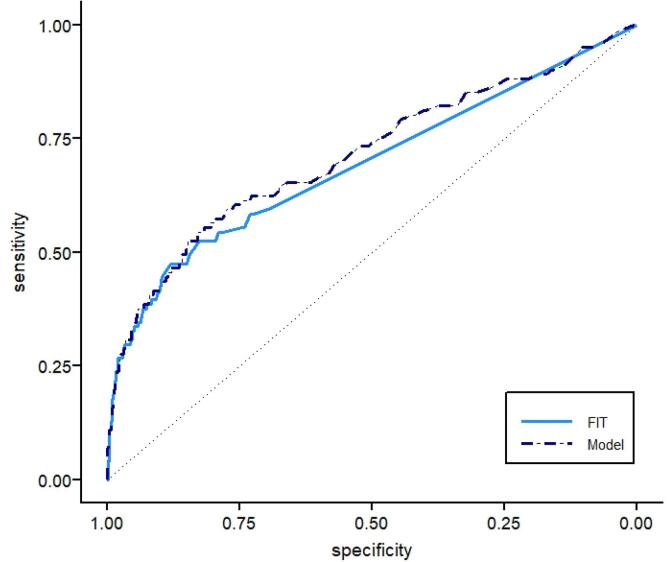
ROC curve of the model and FIT.

The fit of the model with age, FIT and square root of FIT, excluding sex, was significantly better compared to a model with FIT and the square root of FIT only (LR test, *p* = 0.03). Adding sex did not further improve model fit, but since the cost of including the variable sex is negligible and may produce a small gain in terms of prediction, we decided to leave this variable in the model (Table 1). Discrimination of the final model did not significantly improve compared to FIT only: AUC 0.71 (95% CI: 0.65–0.78) vs 0.69 (95% CI: 0.63–0.75) (Fig. 2).

**Table 1:** Coefficients, standard errors, and odds ratios of the model variables (rounded).

Variable	Coefficient	Standard Error	Odds Ratio	95% CI
Intercept	−5.2473	1.1246		
FIT	−0.0141	0.0039	0.99	0.98–0.99
√FIT	0.4555	0.0669	1.58	1.47–1.69
Age (per year)	0.0383	0.0181	1.04	1.003–1.08
Sex	0.0233	0.2250	1.02	0.66–1.59

To show how the risk-based thresholds could perform in clinical practice, we compare performance of the model to that of FIT in two examples. The first example is when a risk-based threshold is chosen that selects an identical number of individuals for colonoscopy, compared to a FIT cut-off concentration of 20 µg Hb/g faeces. With a FIT cut-off at 20 µg Hb/g faeces, the FIT result would be classified as positive in 58 participants in our study group, of which 28 (48.3%) had AN at colonoscopy (CRC *n* = 5, AA *n* = 23). When inviting an identical number of individuals for colonoscopy based on their risk, as calculated with the model, the number of individuals with AN out of those invited for colonoscopy would remain the same (AN *n* = 28, of which CRC *n* = 5 and AA *n* = 23). Using the risk-based thresholds in this scenario would however lead to 12 individuals being reclassified: 6 FIT negative participants would become risk positive, and vice versa (Table 2). The six without AN that would be reclassified as risk positive contains more men (3 versus 1) and has a higher mean age (71 years versus 56.7 years) compared to the six that would be reclassified as risk negative.

**Table 2:** Reclassification table for individuals with and without AN. FIT cut-off concentration is 20 µg Hb/g faeces. The risk positivity threshold (risk = 0.2524) was selected to generate an identical number of positives compared to FIT at 20 µg Hb/g faeces (58 individuals). Using either FIT or risk would lead to detection of AN in 28 of 58 individuals. Twelve individuals would be reclassified.

	FIT positive	FIT negative	Total
*Participants with AN:*			
Risk positive	26	2	28
Risk negative	2	71	73
Total	28	73	101
*Participants without AN:*			
Risk positive	26	4	30
Risk negative	4	977	981
Total	30	981	1011

The second example is when the risk-based thresholds would be used to match the 97% specificity of FIT at a cut-off of 20 µg Hb/g faeces. In this scenario, the model would have returned a positive result for 59 individuals , of which 29 (49.2%) would have AN found (CRC *n* = 5, AA *n* = 24).

### 3.4 Sensitivity and specificity of the risk model

**Fig. 3:**
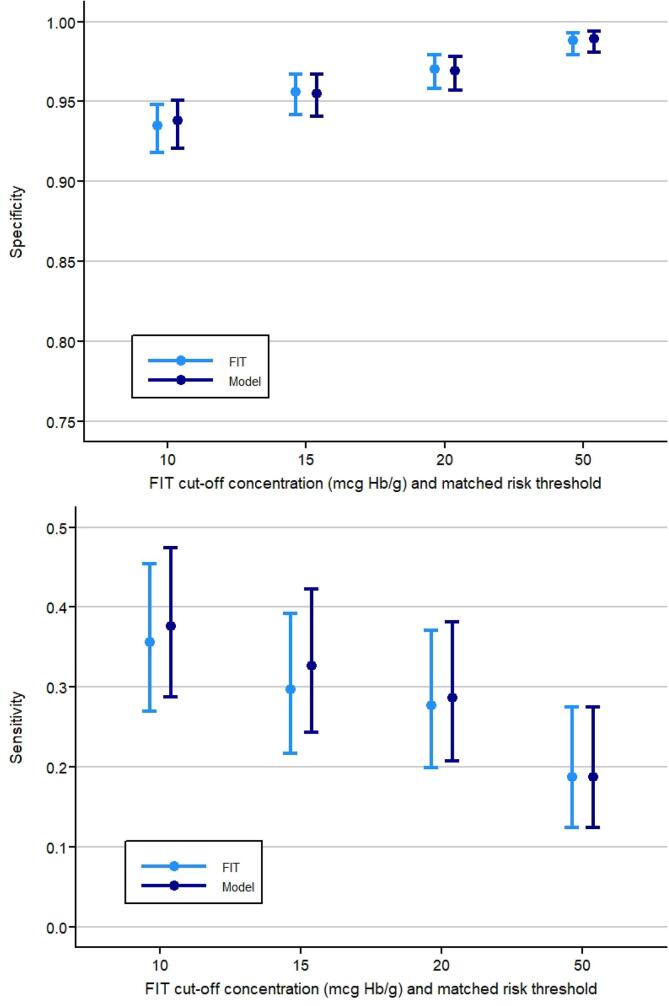
Sensitivity of the FIT and the risk model at different levels of specificity, equivalent to FIT cut-off concentrations of 10, 15, 20, and 50 µg Hb/g faeces. Absolute differences between sensitivities were higher at lower FIT cut-offs and matched risk thresholds.

At a uniform FIT cut-off concentration of 20 µg Hb/g faeces, FIT has a specificity of 97.0% (95%CI: 95.8–97.9) and sensitivity of 27.7% (95%CI: 19.9–37.1) in this study. Matched on specificity, the sensitivity of the risk model was 28.7% (95%CI: 20.8–38.2) at a risk threshold of 0.2524. For men, the specificity and sensitivity at this risk threshold was 96.1% (95%CI: 94.1–97.5) and 30.4% (95%CI: 19.9–43.3) respectively. For women, the specificity was 97.8% (95%CI: 96.1–98.8) and the sensitivity 24.4% (95% CI: 14.2–38.7). For risk thresholds with a similar specificity as FIT at cut-off concentrations of 10, 15 and 50 µg Hb/g faeces, the sensitivity would be 37.6% (95%CI: 28.8–47.4), 32.7% (95%CI: 24.3–42.3), and 18.8% (95%CI: 12.4–27.5) respectively (Fig. 3).

## 4 Discussion

In this study we evaluated a risk model based on data readily available in a screening program and calculated age and sex-based FIT cut-off concentrations necessary to achieve a uniform risk threshold for follow-up colonoscopy. These cut-offs would range from 9.5 to 36.9 µg Hb/g in a risk model with a matched specificity to FIT with a uniform threshold of 20 µg Hb/g. Although there were no statistically significant differences between the sensitivities of different FIT cut-offs and matched risk positivity thresholds, the absolute differences between sensitivities were higher at lower FIT cut-offs. This suggests that models using risk factors such as age and sex may have more benefit at low positivity thresholds.

The effects of age and sex on the risk of CRC or AN have been well described before (Auge et al., 2014, White et al., 2018, McDonald et al., 2011). Other study groups have also explored the use of age, sex, and FIT concentrations in cross-sectional risk models. Auge et al. suggested a model using FIT, age, and sex to prioritize FIT-positive individuals for colonoscopy examination (Auge et al., 2014). In contrast to our results, this group found a significant association between sex and advanced neoplasia. Cubiella et al. developed and externally validated the faecal haemoglobin concentration, age, and sex test (FAST) score in symptomatic patients undergoing colonoscopy (Cubiella et al., 2017). They observed an AUC of 0.79 of the FAST score to detect AN in the external validation cohort (n = 3,976). However, to both studies applies that the pre-test probability of AN in a symptomatic or FIT-positive population is different than that in an asymptomatic average-risk population as targeted in population screening. Although not based on a risk model, Sweden has implemented different FIT cut-offs based on sex with the aim of equalizing cancer detection rates between men and women (Blom et al., 2019). In contrast to our model, which aims to make the risk of having AN identified at colonoscopy in participants comparable, the FIT cut-off concentration in Sweden for women is substantially lower than for men.

A British study investigated the effect of a model based on FIT concentration, age, sex, and participation status in a previous screening round in an average-risk screening population with a FIT concentration of ≥20 µg Hb/g (Cooper et al., 2018). The AUC of the neural network model they developed reached 0.69, significantly higher than the AUC of 0.63 of FIT alone. However, similar to the study of Auge et al., the study was not designed to offer FIT-negative individuals a colonoscopy and therefore missed all false-negative results. In addition, part of the improvement in discrimination can be attributed to data on previous participation. Using FIT concentrations in previous screening rounds or data on previous participation has been proposed as a risk factor for the detection of AN in a present screening round (Cooper et al., 2018, Digby et al., 2017). It must be noted that data on previous participation or previous FIT results are also readily available to screening programs and may achieve better results than our model with similar costs. Since the participants in our study had no previous CRC screening, we could not use such data in our model.

Compared to the studies mentioned above, a strength of our study is that data were collected in a population-based trial where all participants received both FIT and colonoscopy. This enabled us to calculate the sensitivity and specificity of the model for a range of cut-off values. The risk of AN in this study group was comparable to the average risk in the general screening population, since participants were randomly chosen from the general population and individuals with an elevated risk of CRC were excluded beforehand.

A limitation of our study is that the trial was not powered to demonstrate small improvements in AUC, sensitivity, or to explore in differences in sex. In addition, the study was invitation-based and invitees were free to decide whether to participate or not, which may have induced selection bias. The population in our study consisted of individuals without any history of screening who had not undergone a complete colonoscopy within 5 years of study participation.

As many nations have adopted CRC screening, we must acknowledge that most populations have a different risk profile compared to that of our study population. The efficacy of the model may be lower in fully rolled-out screening programs, as older participants often have participated in multiple previous screening rounds and, thereby, have a lower risk of having AN. Adding data from previous screening rounds to the model may be helpful in this situation. We must also note that the data on which the model was developed were collected some time ago. Stegeman et al. used the same dataset to develop a model that reached an AUC of 0.76 (Stegeman et al., 2014). However, this model also used data from a questionnaire, which may affect participation in future applications. In addition, we evaluated individual FIT cut-offs as low as 2.5 µg Hb/g in this study. We are aware that such low cut-offs may not be feasible, because the limit of detection of some FIT assays ranges between 2 and 5 µg Hb/g (Fraser, 2017, Carroll et al., 2014).

One could question whether using risk-based thresholds is more ethical than the current uniform FIT concentration cut-off strategy. While it creates an equal minimal risk of detecting AN in all participants invited for follow-up colonoscopy, the model selects fewer younger and more older participants. Therefore, using this model may ultimately not improve the number of quality adjusted life years (QALYs) gained in a screening program, compared to relying on FIT only, if the improvement in performance is not large enough.

Despite the absence of significant improvement in performance in our study, screening using risk models that combine the FIT result with readily available data may still prove useful, given promising results in comparable evaluations in larger studies (Cubiella et al., 2017, Cooper et al., 2018). Larger studies in which these models are externally validated are needed to confirm this. Future studies should also evaluate combinations of other risk factors, for example including FIT concentrations in previous screening rounds (Chen et al., 2011, Grobbee et al., 2017). Others have proposed to use data from medical records for screening, although privacy rights and regulations may form an obstacle (Cooper et al., 2020).

In conclusion, in this study with data from a population-based multicentre randomized trial, we demonstrated that we can calculate FIT cut-off concentrations that vary with age and sex, based on a uniform risk threshold. This may increase screening yield without increasing costs or adding participation barriers for individuals invited for screening.

## Funding

The COCOS trial has received funding from the Netherlands Organization for Health Research and Development (ZonMW), and from the Center for Translational Molecular Medicine (CTMM).

## CRediT authorship contribution statement

**Tim L. Kortlever:** Methodology, Formal analysis, Writing - original draft, Visualization. **Manon van der Vlugt:** Conceptualization, Writing - review & editing. **Evelien Dekker:** Conceptualization, Writing - review & editing. **Patrick M.M. Bossuyt:** Conceptualization, Writing - review & editing, Supervision.

## Declaration of Competing Interest

The authors declare the following financial interests/personal relationships which may be considered as potential competing interests: Evelien Dekker has endoscopic equipment on loan of FujiFilm, and has received a research grant from FujiFilm. She has received a honorarium for consultancy from FujiFilm, Olympus, Tillots, GI Supply and CPP-FAP and a speakers' fee from Olympus, Roche, GI Supply and Norgine. Besides, she is in the supervisory board of eNose. The other authors declare that they have no competing interests.
