# Supplementary material for: Individualized faecal immunochemical test cut-off based on age and sex in colorectal cancer screening
Source: Prev Med Rep. 2021 Jun 9;23:101447. doi: 10.1016/j.pmedr.2021.101447 (PMC8209662; doi:10.1016/j.pmedr.2021.101447)
Supplement: Supplementary data 1 [file mmc1.docx]

**Supplementary Items**

***Supplementary Figure 1.*** *Individualized FIT cut-off concentrations at a model specificity of 93.8% (equivalent to the specificity of FIT at 10 µg Hb/g faeces).*


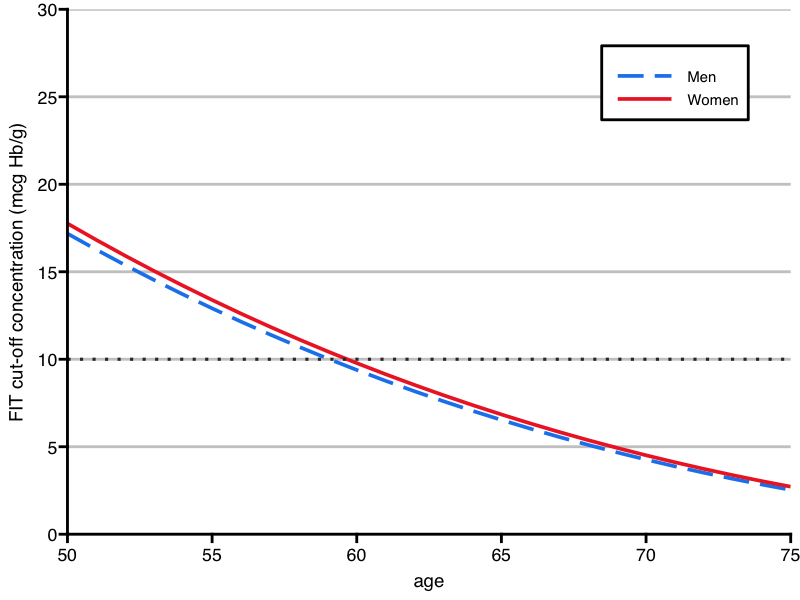


***Supplementary Figure 2****. Individualized FIT cut-off concentrations at a model specificity of 96.9% (equivalent to the specificity of FIT at 15 µg Hb/g faeces).*


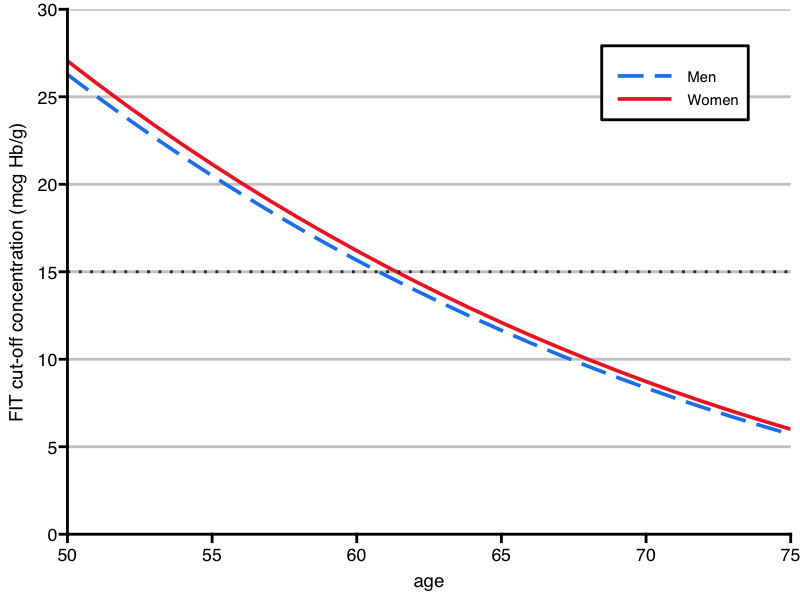


***Supplementary Figure 3****. Individualized FIT cut-off concentrations at a model specificity of 98.9% (equivalent to the specificity of FIT at 50 µg Hb/g faeces).*


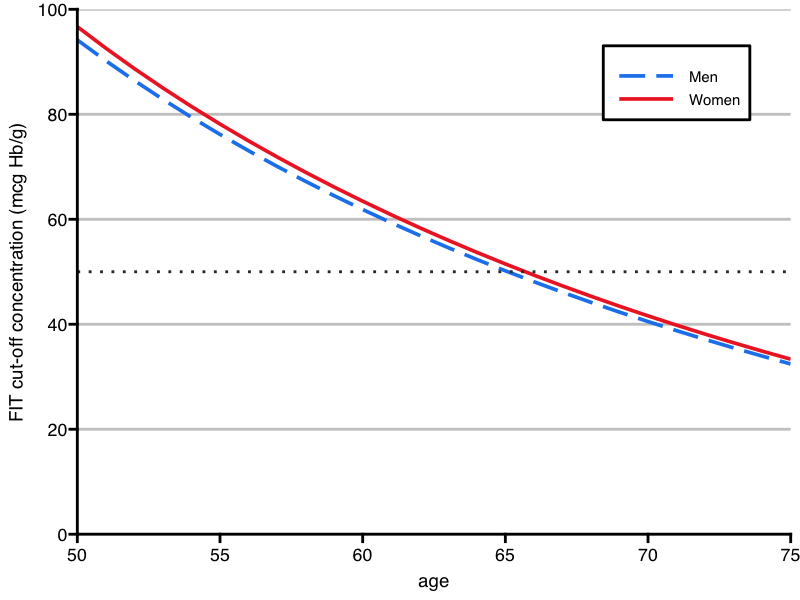


***Supplementary Figure 4****. Individualized FIT cut-off concentrations by age, sex, and model specificity.*

*
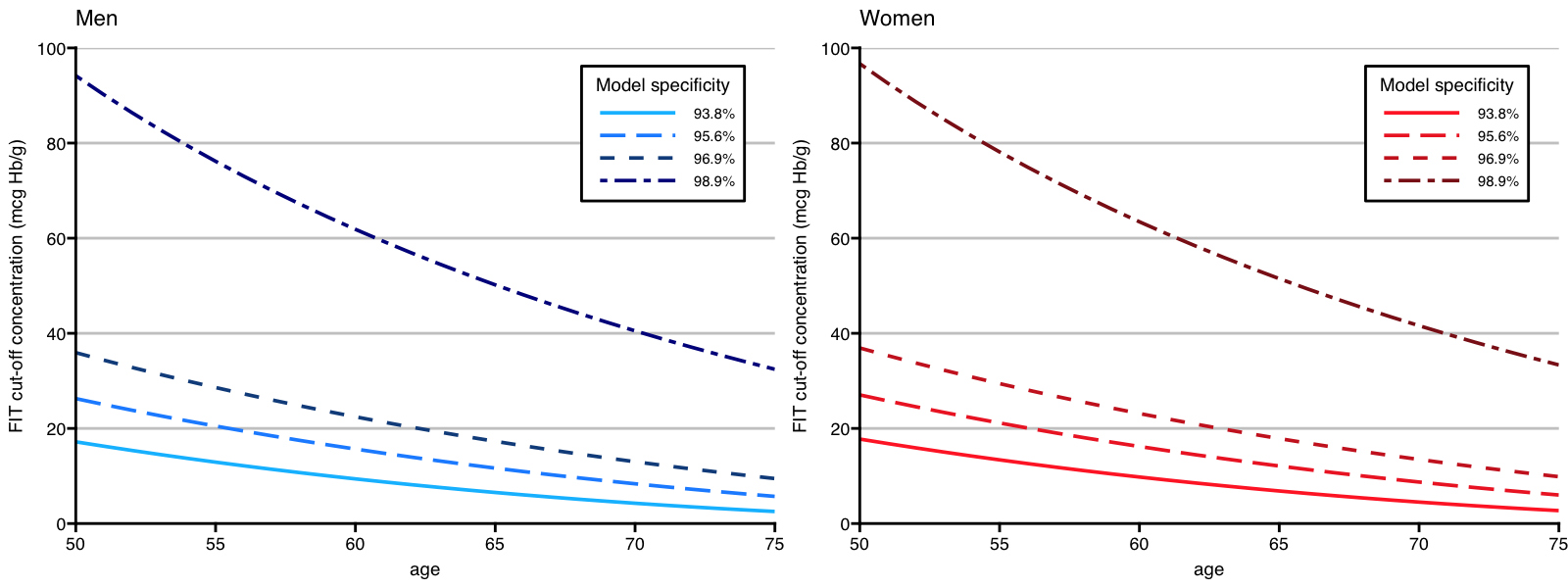
*

***Supplementary Table 1****. Individualized FIT cut-off concentrations at specificities of 93.3%, 95.6%, 96.9%, and 98.9% (equivalent to FIT cut-off concentrations of 10, 15, 20, and 50 µg Hb/g faeces respectively).*

| Age | Specificity = 93.8% | |  | Specificity = 95.6% | |  | Specificity = 96.9% | |  | Specificity = 98.9% | |
| --- | --- | --- | --- | --- | --- | --- | --- | --- | --- | --- | --- |
|  | Men | Women |  | Men | Women |  | Men | Women |  | Men | Women |
| 50 | 17.2 | 17.8 |  | 26.3 | 27.0 |  | 35.9 | 36.9 |  | 94.2 | 96.7 |
| 51 | 16.3 | 16.8 |  | 25.0 | 25.8 |  | 34.4 | 35.3 |  | 90.2 | 92.6 |
| 52 | 15.4 | 15.9 |  | 23.8 | 24.6 |  | 32.8 | 33.8 |  | 86.4 | 88.7 |
| 53 | 14.5 | 15.0 |  | 22.7 | 23.4 |  | 31.4 | 32.3 |  | 82.8 | 85.0 |
| 54 | 13.7 | 14.2 |  | 21.6 | 22.2 |  | 30.0 | 30.8 |  | 79.4 | 81.5 |
| 55 | 12.9 | 13.4 |  | 20.5 | 21.1 |  | 28.6 | 29.4 |  | 76.2 | 78.1 |
| 56 | 12.1 | 12.6 |  | 19.5 | 20.1 |  | 27.3 | 28.1 |  | 73.1 | 74.9 |
| 57 | 11.4 | 11.9 |  | 18.5 | 19.1 |  | 26.0 | 26.8 |  | 70.1 | 71.9 |
| 58 | 10.7 | 11.1 |  | 17.5 | 18.1 |  | 24.8 | 25.5 |  | 67.2 | 68.9 |
| 59 | 10.0 | 10.4 |  | 16.6 | 17.1 |  | 23.6 | 24.3 |  | 64.5 | 66.1 |
| 60 | 9.4 | 9.8 |  | 15.7 | 16.2 |  | 22.4 | 23.1 |  | 61.9 | 63.4 |
| 61 | 8.8 | 9.1 |  | 14.8 | 15.3 |  | 21.3 | 22.0 |  | 59.3 | 60.9 |
| 62 | 8.2 | 8.5 |  | 14.0 | 14.5 |  | 20.3 | 20.9 |  | 56.9 | 58.4 |
| 63 | 7.6 | 7.9 |  | 13.2 | 13.6 |  | 19.2 | 19.9 |  | 54.6 | 56.0 |
| 64 | 7.1 | 7.4 |  | 12.4 | 12.9 |  | 18.2 | 18.8 |  | 52.3 | 53.7 |
| 65 | 6.5 | 6.8 |  | 11.7 | 12.1 |  | 17.3 | 17.9 |  | 50.2 | 51.5 |
| 66 | 6.0 | 6.3 |  | 10.9 | 11.4 |  | 16.4 | 16.9 |  | 48.1 | 49.4 |
| 67 | 5.6 | 5.8 |  | 10.3 | 10.7 |  | 15.5 | 16.0 |  | 46.1 | 47.3 |
| 68 | 5.1 | 5.4 |  | 9.6 | 10.0 |  | 14.6 | 15.1 |  | 44.2 | 45.3 |
| 69 | 4.7 | 4.9 |  | 9.0 | 9.3 |  | 13.8 | 14.3 |  | 42.3 | 43.4 |
| 70 | 4.3 | 4.5 |  | 8.4 | 8.7 |  | 13.0 | 13.5 |  | 40.5 | 41.6 |
| 71 | 3.9 | 4.1 |  | 7.8 | 8.1 |  | 12.2 | 12.7 |  | 38.8 | 39.8 |
| 72 | 3.5 | 3.7 |  | 7.2 | 7.6 |  | 11.5 | 11.9 |  | 37.1 | 38.1 |
| 73 | 3.2 | 3.4 |  | 6.7 | 7.0 |  | 10.8 | 11.2 |  | 35.5 | 36.5 |
| 74 | 2.8 | 3.0 |  | 6.2 | 6.5 |  | 10.1 | 10.5 |  | 33.9 | 34.9 |
| 75 | 2.5 | 2.7 |  | 5.7 | 6.0 |  | 9.5 | 9.9 |  | 32.4 | 33.3 |
